# PPAR Agonists for the Prevention and Treatment of Lung Cancer

**DOI:** 10.1155/2017/8252796

**Published:** 2017-02-20

**Authors:** Sowmya P. Lakshmi, Aravind T. Reddy, Asoka Banno, Raju C. Reddy

**Affiliations:** ^1^Department of Medicine, Division of Pulmonary, Allergy and Critical Care Medicine, University of Pittsburgh School of Medicine, Pittsburgh, PA 15213, USA; ^2^Veterans Affairs Pittsburgh Healthcare System, Pittsburgh, PA 15240, USA

## Abstract

Lung cancer is the most common and most fatal of all malignancies worldwide. Furthermore, with more than half of all lung cancer patients presenting with distant metastases at the time of initial diagnosis, the overall prognosis for the disease is poor. There is thus a desperate need for new prevention and treatment strategies. Recently, a family of nuclear hormone receptors, the peroxisome proliferator-activated receptors (PPARs), has attracted significant attention for its role in various malignancies including lung cancer. Three PPARs, PPAR*α*, PPAR*β*/*δ*, and PPAR*γ*, display distinct biological activities and varied influences on lung cancer biology. PPAR*α* activation generally inhibits tumorigenesis through its antiangiogenic and anti-inflammatory effects. Activated PPAR*γ* is also antitumorigenic and antimetastatic, regulating several functions of cancer cells and controlling the tumor microenvironment. Unlike PPAR*α* and PPAR*γ*, whether PPAR*β*/*δ* activation is anti- or protumorigenic or even inconsequential currently remains an open question that requires additional investigation. This review of current literature emphasizes the multifaceted effects of PPAR agonists in lung cancer and discusses how they may be applied as novel therapeutic strategies for the disease.

## 1. Introduction

Approximately 1.8 million people were newly diagnosed with lung cancer and approximately 1.6 million died from it in 2012, making lung cancer the most common and most fatal malignancy in the world [1]. In the USA alone, 224,390 new cases and 158,080 deaths are estimated for 2016 [2]. A number of risk factors such as hereditary genetic mutations, occupational exposure to lung carcinogens, poor diet, and air pollution have been associated with lung cancer [3]. Chronic lung inflammation and certain pulmonary infections have also shown a positive association [3, 4]. Nevertheless, tobacco smoke is the single, major contributor to the pathogenesis of lung cancer, increasing the lifetime risk even in those who quit smoking. Lung cancer is categorized into two major histological subtypes, small cell lung cancer (SCLC) and non-small cell lung cancer (NSCLC). NSCLC accounts for as much as 85% of all lung cancers and includes adenocarcinoma, squamous cell carcinoma, and large cell carcinoma, while the more aggressive, neuroendocrine tumor SCLC represents most of the rest [5, 6]. Because more than half of lung cancer patients are diagnosed in an advanced stage, with distant metastases [2] and a 5-year survival rate of approximately 2% [7], the overall 5-year relative survival rate for all lung cancer patients combined falls below 18% [2, 7]. This dire state stresses the need for novel approaches in prevention, early detection, and therapy for the disease.

A family of nuclear hormone receptors, the peroxisome proliferator-activated receptors (PPARs), has recently attracted interest as potential therapeutic targets for a variety of malignancies, including lung cancer [8]. Besides being key regulators of lipid and glucose metabolism [9, 10], PPARs, as ligand-activated transcription factors, are also involved in cellular processes including cell differentiation, proliferation, survival, apoptosis, and motility [11–13]. Since many tumors result from dysregulation of these cellular processes and metabolic disorders have been associated with increased cancer risk [14], the role of PPARs in cancer biology is not surprising. PPARs have indeed been implicated in the regulation of various solid cancers as well as leukemias [8, 12].

The PPAR family comprises three members, PPAR*α*, PPAR*β*/*δ*, and PPAR*γ* [15]. Each PPAR subtype is unique in its structure and function [10]. All three PPAR receptors are found in many cells and tissues throughout the body [16–18]. While sharing some common ligands, PPAR family members also respond to distinct repertoires of natural and synthetic ligands, as might be expected from their specific biological activities [13]. PPAR*α* can be activated by fatty acids and eicosanoids (e.g., 8(*S*)-hydroxyeicosatetraenoic acid and leukotriene B_4_) as well as synthetic fibric acid derivatives (e.g., clofibrate and fenofibrate) and pirinixic acid (WY-14,643) [13, 19]. Saturated and unsaturated fatty acids and eicosanoids such as prostacyclin can activate PPAR*β*/*δ* [13, 20]. In addition, synthetic compounds with higher affinities for the receptor have been developed [8, 13]. Natural PPAR*γ* ligands include saturated and unsaturated fatty acids, eicosanoid derivatives such as 15-deoxy-Δ^12,14^-prostaglandin J_2_ (15d-PGJ_2_), and nitrated fatty acids such as nitrated linoleic acid and nitrated oleic acid [21–23]. Synthetic molecules, most notably thiazolidinediones (TZDs) such as pioglitazone, rosiglitazone, troglitazone, and ciglitazone, are potent PPAR*γ* agonists [23]. Upon binding to their respective receptors, these agonists induce dissociation of corepressors that otherwise maintain PPARs in their inactive state [10]. Corepressor dissociation allows the receptors to heterodimerize with retinoid X receptors and initiate transcription by binding to specific PPAR response elements in the promoter regions of their target genes [10]. Emerging evidence suggests that each PPAR regulates tumorigenesis of different cancer types. Moreover, it has been reported that expression of all three isotypes is altered during lung carcinogenesis [24–26]. Thus, PPAR agonists hold potential as novel chemopreventive and therapeutic agents for lung cancer, warranting a review of current literature and further investigation.

## 2. PPARs in Lung Cancer

### 2.1. PPAR*α*

PPAR*α* was the first PPAR subtype to be identified [27]. Its primary function is to regulate energy homeostasis, controlling fatty acid catabolism and lipoprotein metabolism, especially in the liver, as well as metabolism of glucose and amino acids [10, 11, 14].* In vitro* and* in vivo* studies have shown that PPAR*α* agonists also play a regulatory role in inflammatory responses [10]. The function of PPAR*α* during carcinogenesis has not been extensively defined, with most available studies focusing on its role in hepatocarcinogenesis in rodents. In this context, long-term PPAR*α* activation leads to the development of tumors via induction of DNA replication and cell proliferation and suppression of apoptosis [11, 16]. Reactive oxygen species that are byproducts of fatty acid metabolism mediated by PPAR*α* are also thought to contribute to tumorigenesis [11, 16]. Further supporting the involvement of PPAR*α* in hepatocarcinogenesis is the finding that mice lacking PPAR*α* are resistant to the agonist WY-14,643-induced increase in DNA synthesis and formation of hepatic neoplasia [28]. Interestingly, epidemiological data suggest this tumorigenic effect of PPAR*α* activation is absent in humans [11, 16], perhaps due to significantly lower expression of PPAR*α* in human hepatocytes and/or inefficient ligand activation of human PPAR*α* [11]. Another plausible explanation suggests that human PPAR*α* does not exert carcinogenic effects, as activation of a humanized PPAR*α* in transgenic mice does not induce hepatic tumors [14]. In sum, although the between-species variation in effects of PPAR*α* activation on liver carcinogenesis requires further elucidation, humans appear to be protected from the harmful outcomes of PPAR*α* agonists [11].

The involvement of PPAR*α* in lung cancer biology has been extensively investigated within the past decade. A study using a mouse xenograft model showed that absence of PPAR*α* expression in the host animals suppresses tumor growth of Lewis lung carcinoma (LLC) cells and lung and liver metastasis of B16 melanoma cells [29]. This suppression of tumorigenesis and metastasis reflects an increase in leukocyte infiltration of the tumor that is associated with host tissues' antitumor inflammatory responses as well as a reduction in tumor angiogenesis. Intriguingly, the same research group found that PPAR*α* agonists such as fenofibrate and WY-14,643 have the same antitumorigenic and antiangiogenic effects via host PPAR*α* [30]. Together, these two seemingly contradictory observations imply that the antitumor effect of PPAR*α* may be two-pronged; complete absence of PPAR*α* expression allows tumor clearance by the host's immune system while agonist-induced stimulation of PPAR*α* prohibits the exaggerated inflammatory responses of the host that can aggravate tumor development [29, 30].

WY-14,643 has also demonstrated a similar antiangiogenic effect, consequently inhibiting tumor formation in a mouse xenograft model established with A549 NSCLC cells as well as in a mouse model of spontaneous NSCLC [31, 32]. In addition to suppressing primary tumor development, PPAR*α* activation by WY-14,643 inhibits metastasis to the contralateral lung and to the liver in an orthotopic NSCLC model [32]. This negative effect of PPAR*α* stimulation during carcinogenesis is directed toward proliferation of endothelial cells, rather than tumor cells, via suppression of epoxyeicosatrienoic acid biosynthesis [31, 32]. Epoxyeicosatrienoic acids have been shown by both* in vitro* and* in vivo* studies to be proangiogenic [32]. Lastly, fenofibrate treatment was found in mice to significantly abrogate neoplasia formation induced by the potent carcinogen 4-nitroquinoline 1-oxide [33]. These studies supporting the antitumorigenic effect of PPAR*α* agonists, combined with clinical efficacy and safety of these molecules in treating hyperlipidemia, certainly warrant closer investigation of PPAR*α* as a therapeutic target in lung cancer.

### 2.2. PPAR*β*/*δ*

PPAR*β*/*δ* is involved in a variety of physiological processes including embryonic development, lipid metabolism, wound healing, and inflammation [11, 14, 16, 34]. Its critical role in regulation of cellular functions such as adhesion, proliferation, differentiation, and survival has also been well characterized, especially in keratinocytes [11, 16, 34], strongly suggesting its involvement in carcinogenesis. The biological function of PPAR*β*/*δ* in cancer has perhaps been most studied in colon cancer. However, its effect during carcinogenesis remains highly controversial due to lack of consensus in clinical and experimental data. One controversy revolves around expression of PPAR*β*/*δ*, with some studies reporting enhanced expression in colon tumors compared to nontransformed colonic epithelium in which PPAR*β*/*δ* expression is normally high. However, most of these studies are associated with significant limitations such as small sample size, lack of appropriate controls, or inadequate experimental methods and thus need to be interpreted with some caution [14]. The most robust findings to date are provided by the recent retrospective clinical analysis of 141 subjects, showing that higher PPAR*β*/*δ* expression in primary colorectal tumors is associated with lower expression of a marker related to cell proliferation rate, more differentiated cells, reduced rate of lymph node metastasis, and better patient survival following radiation treatment [35]. This report supports the protective role of PPAR*β*/*δ* in human colorectal cancer. In contrast, an* in vivo* study using a colorectal cancer cell xenograft model found that PPAR*β*/*δ* deficiency in the grafted tumor cells suppresses tumor growth, suggesting a protumorigenic role [36]. However, when interpreting these expression data, it is important to note that PPAR*β*/*δ* expression does not indicate the receptor is functionally active; the receptor's activity can be modulated by a variety of factors such as ligand availability and the presence or absence of other proteins [14] as well as by posttranslational modifications. Thus, future studies should examine and compare activity state in addition to expression of PPAR*β*/*δ* in tumors and normal tissue counterparts.

Evidence regarding functional outcomes of PPAR*β*/*δ* activation is similarly contradictory; some studies show activated PPAR*β*/*δ* promotes tumor development by stimulating cell proliferation and preventing apoptosis while others propose receptor activation attenuates tumorigenesis by inducing differentiation and suppressing exaggerated inflammatory responses [14, 16]. Several molecular mechanisms have been proposed to underlie PPAR*β*/*δ*'s effect on tumorigenesis. Two pathways implicated in its protumorigenic effect are increased expression of vascular endothelial growth factor (VEGF) and enhanced prosurvival signaling involving integrin linked kinase (ILK), 3-phosphoinositide-dependent-protein kinase 1 (PDPK1), phosphatase and tensin homolog deleted on chromosome 10 (PTEN), and AKT [14]. It has been shown that the antitumorigenic effect is mediated through enhanced activity of prodifferentiation genes and/or suppression of proinflammatory signals mediated primarily by the NF-*κ*B pathway [14].

Similar discrepancies are present in cancers of other tissues, with the exception of skin cancer where there seems to be general agreement on the protective role of PPAR*β*/*δ* [14]. The involvement of PPAR*β*/*δ* in lung cancer was first reported in an* in vitro* study showing that the agonist L-165041 induces growth inhibition of A549 cells, as evidenced by decreased expression of the proliferation marker proliferating cell nuclear antigen (PCNA) [37]. The authors identified induction of G_1_ cell cycle arrest as a result of reduced cyclin D expression, rather than induction of apoptosis, as the underlying mechanism. This antiproliferative effect of PPAR*β*/*δ* activation parallels several* in vivo* observations. Although involvement of PPAR*β*/*δ* was not directly assessed and a PPAR*β*/*δ*-independent mechanism remains a viable possibility, one research group used multiple lung cancer models to demonstrate that lung tumorigenesis is suppressed by increased synthesis of the PPAR*β*/*δ* agonist prostacyclin [38, 39]. Mice lacking PPAR*β*/*δ* expression also display increased tumor incidence in a RAF-induced lung cancer model [40]. These* in vitro* and* in vivo* data suggest a protective role of PPAR*β*/*δ* against lung cancer.

It has also been postulated that PPAR*β*/*δ* may prevent lung cancer via its anti-inflammatory function, as it does with colorectal cancer. In two independent studies, one using a lipopolysaccharide-induced pulmonary inflammation model [41] and the other using a carrageenan-induced pleurisy model [42], the potent PPAR*β*/*δ* agonist GW0742 was shown to reduce neutrophil infiltration into the lungs and suppress expression of proinflammatory cytokines such as interleukin-6 (IL-6), IL-1*β*, and tumor necrosis factor-*α* (TNF-*α*) [41, 42]. Although these studies did not assess the effect of PPAR*β*/*δ*'s anti-inflammatory activity on lung carcinogenesis, pulmonary inflammation has been implicated as a contributing factor [4, 12, 43]. The anti-inflammatory function of PPAR*β*/*δ* agonists in the context of lung cancer biology is therefore worthy of further investigation.

In contrast to these studies, however, others have provided evidence that PPAR*β*/*δ* activation promotes lung cancer; the agonist GW501516 stimulates proliferation, inhibits apoptosis, and supports anchorage-independent growth of A549, H157, and H23 NSCLC cells [25]. The proliferative effect is mediated through PDPK1 overexpression, increased AKT phosphorylation, and PTEN suppression, while resistance to apoptosis results from enhanced expression of B-cell lymphoma-extra large (Bcl-x_L_) and cyclooxygenase-2 (COX-2). PPAR*β*/*δ* can potentiate tumor formation by modulation not only of cancer cells but also of nontransformed cells in the tumor microenvironment. In a mouse xenograft model with LLC cells, absence of PPAR*β*/*δ* in the host animals significantly reduced tumor volume and improved survival of the animals [44]. This suppression of LLC cell tumor growth in the PPAR*β*/*δ*-deficient mice is a consequence of dysregulated angiogenesis and reduced blood flow.

Other studies suggest that PPAR*β*/*δ* may not influence tumorigenesis at all. One such study observed that GW501516 or GW0742 had no effect on expression of PTEN or PDPK1, or on AKT phosphorylation, in A549 or H1838 NSCLC cells, implying that PPAR*β*/*δ* activation does not influence these cells' proliferation [45]. No change in the percentage of cells in each phase of the cell cycle was observed either. Likewise, the PPAR*β*/*δ* antagonist GSK3787 did not affect proliferation of A549 or H1838 cells [46].

Thus, based on our current knowledge, it is difficult to draw a definite conclusion regarding the biological effect of PPAR*β*/*δ* activation in lung cancer. There are several possible explanations for these discrepancies, however. First, the contradictory results may be related to PPAR*β*/*δ*'s ability to repress as well as induce target gene expression; it has been observed that PPAR*β*/*δ* can repress the transcription of its target genes when not bound by its ligands, whereas ligand-bound PPAR*β*/*δ* induces expression [16]. Secondly, PPAR*β*/*δ* activity may be affected by the presence or absence of cofactors and repressors [16]. Therefore, it is conceivable that the between-study variability in cell culture conditions and genetic background of model animals creates differential cellular environments and thereby leads to the contradictory observations [16]. Finally, many PPAR ligands demonstrate PPAR-dependent and -independent activities, which makes data interpretation more challenging [16]. In summary, further careful analyses are required to delineate the complexities of PPAR*β*/*δ* expression and activation in lung cancer.

### 2.3. PPAR*γ*

PPAR*γ* is an established regulator of adipocyte differentiation, glucose metabolism, and lipid homeostasis [10, 23]. Its involvement in inflammation has also been recognized [10]. More recently, PPAR*γ*'s role in cancer has become apparent; PPAR*γ* hinders tumor development and progression, in most cases by modulating differentiation, proliferation, apoptosis, and motility of cancer cells through a variety of molecular pathways [8, 17, 47, 48]. In addition to regulating the oncogenic activities of cancer cells, PPAR*γ* can control the tumor microenvironment; the receptor creates a hostile environment for tumor growth and metastasis via multiple mechanisms [8, 17]. In the context of lung cancer, with general agreement on its role as a tumor suppressor, the biological effects of activated PPAR*γ* are perhaps better defined than those of PPAR*α* or PPAR*β*/*δ* [8]. As its influence on cancer cells has been extensively reviewed elsewhere [8, 17, 18], this review will focus on how the microenvironment may be affected by PPAR*γ*'s anti-inflammatory function.

Lung carcinogens such as tobacco smoke and inhaled asbestos are known to cause chronic pulmonary inflammation that is associated with lung carcinogenesis [4, 43]. Key players in the cancer-associated inflammatory responses are cellular constituents of the tumor microenvironment such as tumor-associated macrophages, neutrophils, and fibroblasts that secrete growth factors, cytokines, chemokines, reactive oxygen species, and matrix metalloproteinases (MMPs) [4]. The influence of the resulting inflammatory microenvironment on tumor formation and metastasis is multifold and often involves the NF-*κ*B signaling pathway [4]. For instance, inflammation has been shown to increase the rate of genetic mutation within the adjacent epithelial cells and the proliferation of those mutated cells [4]. Tumor-associated macrophages can facilitate tumor angiogenesis, a process that supplies microscopic tumors with nutrients and provides cancer cells with ready access to the circulation required for metastasis [4, 49–51]. The cells mediating cancer-associated inflammatory responses also promote metastasis by contributing MMPs, the key regulator of extracellular matrix remodeling and disruption [4, 52].

PPAR*γ* has been shown to affect multiple aspects of these cancer-associated inflammatory responses. Agonist activation of PPAR*γ*, whose expression increases upon macrophage and monocyte activation [10], suppresses these leukocytes' production of inflammatory mediators such as inducible nitric oxide synthase, MMP-9, scavenger receptor A, TNF-*α*, IL-1*β*, and IL-6 [53–55]. Importantly, these inflammatory molecules have been shown to promote tumorigenesis in several cancers [56]. The negative regulation of inflammatory responses is mediated by the inhibition of transcription factors, for example, NF-*κ*B, activator protein-1 (AP-1), members of the signal transducer and activator of transcription (STAT) protein family, and nuclear factor of activated T cells (NFAT), often via a mechanism termed transrepression [17, 57]. During transrepression, PPAR*γ* interacts with transcription factors and sequesters them from their response elements, preventing inflammatory responses [57]. PPAR*γ* also regulates pathways essential to expression and activity of these transcription factors.

15d-PGJ_2_, troglitazone, ciglitazone, and rosiglitazone [58, 59], as well as constitutively active PPAR*γ* [59], also suppress differentiation of human lung fibroblasts into myofibroblasts [58, 59]. Myofibroblasts within the tumor microenvironment are the predominant source of tumor-supporting extracellular matrix and also produce molecules that facilitate tumor growth and progression [17, 60] and are considered more carcinogenic than normal fibroblasts [60]. PPAR*γ* agonists also prevent the myofibroblast-associated increase in collagen secretion [58, 59] that can result in remodeling of the tumor microenvironment and facilitate cancer pathogenesis [61]. Furthermore, PPAR*γ* agonists demonstrate suppressive effects on neutrophils' chemotactic response and neutrophil cytokine production [62]. As predicted by this concept, in a mouse model of pulmonary inflammation, endothelial cell PPAR*γ* deficiency enhanced neutrophil infiltration into the lungs and exacerbated tissue injury [63]. These studies, providing a link between PPAR*γ*, inflammation, and cancer, highlight the significance of inflammation-associated cells as a trigger of tumorigenesis as well as of PPAR*γ* as a tumor suppressor acting via multiple mechanisms.

It may prove beneficial to pursue PPAR*γ* activation as a novel chemopreventive strategy [64]. The chemopreventive effects of PPAR*γ* agonists in lung cancer have been reported by several studies. Troglitazone and pioglitazone as well as sulindac sulfide, a nonsteroidal anti-inflammatory drug known to activate PPAR*γ*, significantly reduce primary tumor formation by A549 cells in a xenograft mouse model [65, 66]. Pioglitazone also decreases tumor volume and significantly deters disease progression in mouse models of spontaneous lung adenocarcinoma and squamous cell carcinoma induced by vinyl carbamate and N-nitroso-tris-chloroethylurea, respectively [67]. These findings suggest that PPAR*γ* agonists can inhibit epithelial cell transformation in the early stages of tumorigenesis. Most significantly, one epidemiologic analysis of diabetic patients from 10 Veterans Affairs medical centers, comparing 11,289 TZD users with 76,389 nonusers, observed a 33% reduction in subsequent lung cancer diagnosis in the former group [68], thus underscoring the chemopreventive potential of PPAR*γ* agonists. A clinical trial (NCT00780234) designed to assess the ability of pioglitazone to prevent lung cancer in a more general, nondiabetic population has been initiated, and its results may provide additional justification for the application of PPAR*γ* agonists as a chemopreventive strategy against lung cancer.

## 3. Therapeutic Application of PPAR Ligands for Lung Cancer

All three members of the PPAR family demonstrate involvement in carcinogenesis, although their mode of action differs. These receptors are therefore attractive targets for lung cancer prevention and treatment. Indeed, the therapeutic applicability of PPAR*γ* agonists is evident in several studies. Besides experimental data supporting the use of PPAR*γ* agonists as a monotherapy for lung cancer, as discussed above, multiple PPAR*γ* agonists demonstrate synergy with commonly used traditional chemotherapeutic drugs such as cisplatin, carboplatin, and paclitaxel, inhibiting proliferation of multiple NSCLC cell lines and suppressing tumor growth in a xenograft lung cancer model [69, 70]. This synergistic effect has also been observed between PPAR*γ* agonists and targeted therapies such as gefitinib, an epidermal growth factor receptor inhibitor, and lovastatin, an inhibitor of 3-hydroxy-3-methyl-glutaryl-coenzyme A reductase (HMG-CoA reductase) [71, 72]. These data substantiate the chemotherapeutic potential of PPAR*γ* agonists. Unlike PPAR*γ* agonists, however, the clinical applicability of PPAR*α* and PPAR*β*/*δ* ligands in lung cancer has not been assessed. Nevertheless, the PPAR*α* fibrate agonists have proven relatively safe and effective for treatment of dyslipidemia and cardiovascular disease [11] and clinical assessment of PPAR*β*/*δ* ligands should be starting soon. In this context, reported studies showing their physiological effects in lung cancer make a strong argument for further investigation of their chemopreventive and chemotherapeutic potentials.

Some PPAR agonists activate two or all three PPAR receptors [14, 73]. Recently, an intriguing concept has emerged suggesting that use of these dual- or pan-PPAR agonists may be more beneficial than using agents targeting a single PPAR subtype. For instance, one recent clinical trial observed bezafibrate, a pan-PPAR agonist, reduced development of new colon cancer by 53%, although there was no comparison to more selective PPAR agonists [74]. This finding can be interpreted as showing that lower-affinity pan-PPAR agonists may be useful as a novel chemopreventive strategy [14]. Simultaneous activation of PPAR*α* and/or PPAR*β*/*δ* may also alleviate the known side effects of PPAR*γ* agonists (weight gain and bone fractures) by stimulating lipid metabolism and bone formation [14, 73]. Thus, activation of more than one PPAR receptor should also be pursued as a new therapeutic approach in lung cancer.

## 4. Conclusions

Our understanding of PPARs in lung cancer remains incomplete. In particular, the effect of PPAR*β*/*δ* expression and activation on carcinogenesis has yet to be delineated and the controversy regarding its role must be resolved before the potential of its agonists/antagonists as therapeutic agents can be evaluated. Nevertheless, based on review of extensive experimental data, the involvement of all three PPAR receptors in lung cancer biology is undeniable. Therefore, with further investigation and additional clinical trials, PPAR modulators may become a valuable tool in the prevention and treatment of lung cancer.

## Abbreviations

15d-PGJ_2_: 15-Deoxy-Δ^12,14^-prostaglandin J_2_
AP-1: Activator protein-1
Bcl-x_L_: B-cell lymphoma-extra large
COX-2: Cyclooxygenase-2
HMG-CoA reductase: 3-Hydroxy-3-methyl-glutaryl-coenzyme A reductase
IL: Interleukin
ILK: Integrin linked kinase
LLC: Lewis lung carcinoma
MMP: Matrix metalloproteinase
NFAT: Nuclear factor of activated T cells
NSCLC: Non-small cell lung cancer
PCNA: Proliferating cell nuclear antigen
PDPK1: 3-Phosphoinositide-dependent-protein kinase 1
PPAR: Peroxisome proliferator-activated receptor
PTEN: Phosphatase and tensin homolog deleted on chromosome 10
SCLC: Small cell lung cancer
STAT: Signal transducer and activator of transcription
TNF-*α*: Tumor necrosis factor-*α*
TZD: Thiazolidinedione
VEGF: Vascular endothelial growth factor.
